# Effects of Different Spectral Energy Distributions on Physiological Behavior and Hormone Levels in Depression

**Published:** 2018-03

**Authors:** Chunyu YANG, Zhiyuan ZHANG, Juntao MA, Ting CHEN

**Affiliations:** Faculty of Architecture and Urban Planning, Chongqing University, Chongqing, China

**Keywords:** Depressive symptoms, Artificial light spectrum, Behavioristics, Monoamine neurotransmitter

## Abstract

**Background:**

The increasing worldwide incidence of depression causes massive economic losses to the country and society. Insufficient sun exposure aggravates depressive symptoms in patients with depression. Preventive light replenishment is provided to patients with depression and the potential population. In addition, we studied the applicable spectrum, which is crucial in the prevention of depression.

**Methods:**

Forty depressed male rats were randomly divided into five groups of 8 rats each: depressive model, microwave sulfur lamp, halogen lamp, fluorescent lamp, and LED lamp groups. Rats in the illuminated groups were exposed to light for 45 days for 2 h daily. Eight healthy rats were selected for the control group. The body weight and general behaviors of rats were recorded. After the experiment, peripheral blood was collected from the tail vein, and the concentrations of MT, 5-HT, NA, and BDNF in serum were detected by ELISA.

**Results:**

After the model was established, the body weight of rats in the depressive model group increased slowly. Compared with those of the control group, the results of the three behavioral tests were significantly different (*P*<0.05); the contents of MT, 5-HT, NA, and BDNF were relatively low (*P*<0.05). In addition, depression characteristics were significant. Rats regained their pleasant sensation after microwave sulfur lamp intervention. Compared with the rats in the depressive model group, the levels of MT, 5-HT, NA, and BDNF increased sharply.

**Conclusion:**

The spectral energy distribution of microwave sulfur lamp is similar to the solar spectrum, which can alleviate depressive symptoms in depressed rats.

## Introduction

Depression is a typical mental disorder that seriously affects public health and causes massive social losses. At present, the incidence of depression worldwide is about 11%, making it the fourth major disease globally. By 2020, depression is expected to become the world’s second most common disease, following coronary heart disease (1). More than 26 million people suffer from depressive disorder in China, with an average of 240 000 people committing suicide each year because of depression. The total economic burden caused by depression reached 62.2 billion CNY. For a long time, patients with depression have been predominantly drug treated. Professor Holsboer, the director of the Max Planck Institute for Psychiatry, argued that decades of clinical drug treatment yield side effects, and the results are frustrating (2). Either major depressive disorder (3) or seasonal affective disorder, which are subtypes of depression (4–5), aggravates symptoms owing to limited solar environment. According to one of the most important hypotheses to treat depression by using single or combined phototherapy, the level of melatonin in the body by supplementary light affects the biological rhythm and relieves symptoms (6–9). In 2010, the revised Guidelines for the Treatment of Depressed Patients (Third Edition) by the American Psychiatric Association included phototherapy in the therapeutic regimen (10).

Human biological clock system features photoreception, and abnormal night light inhibits the secretion of melatonin (11). Subsequently, depression can be treated by artificial light (12). The third type of photoreceptor cell, ipRGCs was reported in the USA (13). Visible radiation participates in the regulation and control of human vital sign changes, such as hormone secretion and excitement degree, by ipRGCs, which affect human physiology and psychology. Rosenthal et al. proved that light can effectively alleviate the depressive symptoms of patients with depression (14).

Compared with traditional drug treatment, phototherapy presents good economic performance and no anticholinergic side effects. Phototherapy after onset is inappropriate; they also stated that protective phototherapy is an effective method (15). The antidepressant effect of phototherapy is related to lighting parameters, such as spectrum, light intensity, and duration. At present, numerous light treatments for depression are available, with intensity ranging from 100 lux to 10000 lux. In terms of spectrum, treatments can involve the monochromatic spectra, including red and blue light, with wavelength ranging from 470 nm to 670 nm, which includes the composite spectrum of white light. Either monochromatic spectrum or composite spectrum of white light featured by high illuminance is common in clinical treatment and the effects after lighting is varied (16–18). Few reports are available on the protective phototherapy indoor properties. Additional studies have focused on the relaxation effect of different light intensities on depression. The exact effects of phototherapy with different spectral energy distribution on depression treatment will be explored (19).

The theoretical basis of the depressive rat model of chronic unpredictable mild stress (CUMS) is close to that of human depression. Thus, the model can be adopted to simulate core symptoms, such as lack of pleasant sensation, decline in sporting and social skills, and decline in the capability of exploratory behavior.

To study the effects of supplementary illumination with different spectral energy distributions on human depression, we established a CUMS depressive rat model in the experiment (cultured by the Neuroscience Research Center of Chongqing Medical University). A total of four indoor artificial light spectra were selected for light interference. The halogen lamp features a continuous spectrum and long-wave radiation. The florescent lamp and LED lamp feature discontinuous spectrum with energy mainly concentrated on blue–green light. The full-spectrum microwave sulfur lamp features a continuous spectrum and closely simulates the solar spectrum. The effects of artificial illumination with different spectral energy distributions on physiological behavior and hormone concentrations in vivo of depressed rats were observed.

## Materials and Methods

### Animals

A total of 48 adult male Sprague–Dawley rats with body weights ranging from 230 g to 270 g (Experimental Animal Center of Chongqing Medical University, production license number: SGKK [Yu] 2005-0001 were included in the experiment. The study was approved by Ethics Committee of Chongqing Medical University and was conducted according to the declaration of Helsinki.

The feeding conditions complied with the following relevant requirements of GB14925-2010 “laboratory animal environment and facilities”: Specific Pathogen Free(SPF)-shielded ambient temperature between 20 °C and 25 °C, and relative humidity between 50% and 70%, and artificial circadian rhythm of 12 h (08:00 to 20:00). Rats were housed separately in cages and were free to take food and drink water.

### Reagents and instruments

**Fig. 1:**
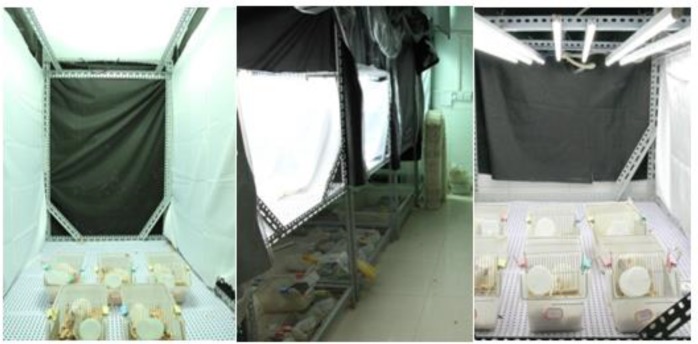
Experimental lamps and scene

**Fig. 2:**
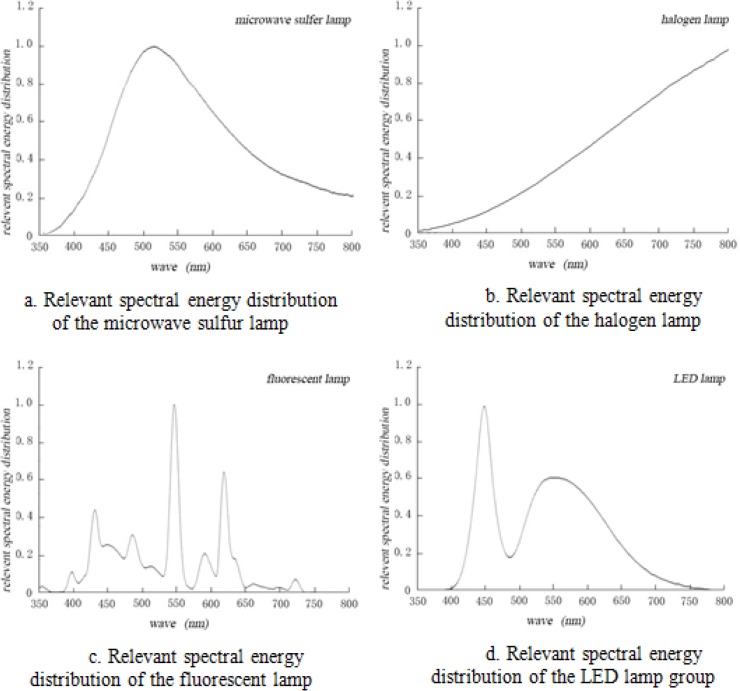
Relevant spectral energy distributions of four artificial light sources

The instruments used in the experiment were as follows: SPF-graded rat feed for the maintenance period; analysis system of animal behavior; spontaneous activity-based video analysis system; electronic scales; camera; microcomputer time switch; ELISA kit (Shanghai Jimian Biotechnology Co., Ltd.); vacuum coagulating blood collection tube (Shanghai Kehua Testing Medical Products Co., Ltd.); Konica CL-500A spectroradiometer; PSH0731B plasma microwave sulfur lamp by LG (peak value: 510 nm; illuminance: 3000 lux; color temperature: 6900 K; horizontal uniformity: 0.82); Fluorescent lamp (peak value: 550 nm; illuminance: 3000 lux; color temperature: 6500 K; horizontal uniformity: 0.84); LED lamp (peak value: 450 nm; illuminance: 3000 lux; color temperature: 6500 K; horizontal uniformity: 0.80); Halogen lamp (illuminance: 1100 lux; color temperature: 2466 K; horizontal uniformity: 0.76). The experimental site and the relative spectral energy distribution of the illumination groups are shown in Fig. 1 and 2.

### Methods

#### Establishment of the CUMS depressed rat model

After 1 week of adaptive feed, 48 laboratory rats were subjected to open-field (horizontal movement score + vertical movement score), forced swimming (dead time), and sucrose preference tests (24-h sucrose preference). Afterward, the experimental data were recorded. Then, 8 rats were randomly selected and included in the control group (without any stress), whereas the remaining 40 rats were randomly divided into five groups with 8 rats in each group. The five treatment groups include depressive model (without light treatment), microwave sulfur, halogen, fluorescent, and LED lamp groups. Afterward, the rats were subjected to a total of 10 unpredictable mild stress: water deprivation, feeding with ambrosia, swimming in icy water, day and night reversal, tail clipping, electrophotoluminescence, 45° tilting of the rat cage, horizontal vibration, overexposure to the sun’s rays, and wet padding. Stress treatment lasted for 35 days, with each stress applied for 3 times to 4 times, and the general behavioral observation and recording were performed simultaneously. After 35 days, open-field, forced swimming, and sucrose preference tests were performed again. Compared with the rats in the control group, the five rat groups displayed significant difference (*P*<0.05), indicating the successful modeling of the depressed rats.

#### Illumination experiment

During 12:00 to 14:00 daily, rats in the microwave sulfur, fluorescent, LED, and halogen lamp groups were placed in lighted environments with different spectra, and rats in the depressive model group remained untreated. General behavior of rats was observed and recorded daily. For 45 consecutive days, room temperature was between 24 °C and 26 °C, and the relative humidity was between 65% and 66%.

#### Test of hormone and neurotransmitter in the serum

After the illumination experiment was completed, peripheral blood was collected from the tail vein of rats, and the liquid supernatant was collected after centrifugation. Liquid supernatants were placed in a −80 °C low-temperature refrigerator. Through ELISA, serotonin (5-HT), melatonin (MT), norepinephrine (NA), and brain-derived neurotrophic factor (BDNF) concentrations in the serum were obtained and tested by Shanghai Jimian Biotechnology Co., Ltd. and transported in dry ice.

#### Statistical treatment

Experimental data were analyzed by SPSS 22.0 statistical software (Chicago, IL, USA). Data were presented as mean±SD. The tests show that the data of each group is subject to normal distribution, with homogeneity of variance. One-way ANOVA (20) was adopted for analysis, and SNK-q test was used for pair-wise comparisons. Statistical significance was determined with *P* value of <0.05.

## Results

### Growing Condition

After modeling, rats in the depressive model group were limited in terms of food ration. The rats showed low spirits and slow response. In addition, the times of grooming were reduced, with slow growth of body weight relative to the control group. After supplementary illumination, the rats in the microwave sulfur lamp group displayed significant differences in body weight (*P*=0.029) compared with rats in the depressive model group, whereas rats in the control group did not display significant difference in body weight. Rats in other illumination groups displayed significant differences from the rats in the control group (Table 1).

**Table 1:** Body weights of rats in each group before stress, after stress, and after light illumination (mean±SD; n=8)

***Group***	***Before stress***	***35 days after stress***	***45 days after light***
Control	268.67±7.51	303.50±36.06	422.12±20.73
Depressive model	248.59±51.92	275.81±38.66	311.00±23.36^#^
Microwave sulfur lamp	271.13±63.73	277.57±39.48	362.33±57.00^*^
Halogen lamp	264.38±65.58	285.00±53.65	323.87±43.52^#^
Fluorescent lamp	237.29±47.29	260.43±28.26	354.40±29.07^#^
LED lamp	232.25±44.15	267.63±45.32	331.83±34.97^#^

Note:

# *P*<0.05 vs control group;

* *P*<0.05 vs depressive model group

### Results of behavioristics experiment

After modeling, the rats in the remaining groups displayed significant differences compared with the control group in terms of scores in the open-field experiment, sucrose preference, and dead time of forced swimming; these differences indicated successful modeling. After lighting, three sets of data of rats in the illumination groups showed increases.

Among the groups, the LED lamp group rats showed significant differences (*P*=0.031) compared with those in the depressive model group in terms of sucrose preference. However, the same group did not display significant difference compared with the control group. Rats in the microwave sulfur lamp group showed significant differences in three data sets (*P*=0.028; *P*=0.040; *P*=0.001) compared with the rats in the depressive model group but did not show significant difference from the control group (Table 2–4).

**Table 2:** Data on rats in each group before stress application, after stress application, and after light illumination in the open-field tests (mean±SD. n=8)

***Group***	***Number of crossings***		
	**Before stress**	**After stress**	**After lighting**
Control	128.43±21.67	125.50±19.76	123.50±20.92
Depressive model	119.37±24.15	55.37±27.49^#^	49.33±28.01^#^
Microwave sulfur lamp	124.37±16.44	57.25±32.64^#^	98.80±19.01^*^
Halogen lamp	136.50±19.60	46.87±48.01^#^	70.12±43.34^#^
Fluorescent lamp	132.62±22.02	55.00±34.59^#^	73.40±42.79
LED lamp	120.71±12.02	44.12±47.60^#^	70.00±45.41^#^

Note:

# *P*<0.05 vs control group;

* *P*<0.05 vs depressive model group

**Table 3:** Data on rats in each group before stress application, after stress application, and after light illumination in the sucrose preference tests (mean±SD. n=8)

***Group***	***Percentage of sucrose consumption (%)***		
	**Before stress**	**After stress**	**After light**
Control	94.89±2.31	95.39±2.56	96.52±3.47
Depressive model	95.57±2.51	80.61±7.02^#^	80.26±6.31^#^
Microwave sulfur lamp	95.81±1.86	78.00±6.16^#^	92.25±5.80^*^
Halogen lamp	93.29±5.77	76.25±15.63^#^	86.21±4.75^#^
Fluorescent lamp	95.98±1.60	74.71±9.84^#^	87.29±9.69^#^
LED lamp	94.39±3.27	76.00±9.61^#^	93.19±1.43^*^

Note:

# *P*<0.05 vs control group;

* *P*<0.05 vs depressive model group

**Table 4:** Data on rats in each group before stress application, after stress application, and after light illumination in the forced swimming tests (mean±SD. n=8)

***Group***	***Immobility times(s)***		
	**Before stress**	**After stress**	**After light**
Control	6.28±4.42	5.87±4.64	7.35±7.84
Depressive model	5.33±5.37	49.96±24.98^#^	43.00±27.30^#^
Microwave sulfur lamp	5.15±6.84	33.60±16.78^#^	9.81±10.42^*^
Halogen lamp	9.51±5.77	40.44±28.66^#^	28.76±20.85^#^
Fluorescent lamp	8.30±13.21	32.68±21.65^#^	29.80±11.73^#^
LED lamp	5.88±5.79	30.90±12.60^#^	26.97±9.08^#^

Note:

# *P*<0.05 vs. control group;

* *P*<0.05 vs. depressive model group

### Results of the illumination experiment with different spectra

In the experiment, MT, 5-HT, and NA monoamine neurotransmitters and BDNF acted as assessment indicators for the effects of different spectra on depressed rats. A total of four serum concentrations of depressed rat hormones were significantly decreased compared with those of control rats. After light illumination, the MT (*P*=0.023), 5-HT (*P*=0.018), NA (*P*=0.039), and BDNF (*P*=0.038) concentrations in the microwave sulfur lamp group increased again. Significant differences were observed between the microwave sulfur lamp and the depressive model group, demonstrating relatively good consistency. Compared with those in the depressive model group, the concentrations of the four hormones in other illumination groups again increased to a certain level. The NA in the LED group (*P*=0.030) and BDNF in the fluorescent lamp group (*P*=0.014) displayed significant differences, (Table 5).

**Table 5:** MT, 5-HT, NA, and BDNF concentrations in rat serum from each group (ng/L, mean±SD, n=8)

***Group***	***MT (ng/L)***	***5-HT (ng/L)***	***NA (ng/L)***	***BDNF (ng/L)***
Control	30.17±5.92	283.88±26.54	136.77±15.44	106.60±11.41
Depressive model	24.87±3.46^#^	224.74±11.43^#^	116.81±24.23^#^	87.93±9.14^#^
Microwave sulfur lamp	30.37±3.19^*^	288.85±43.66^*^	137.81±17.51^*^	103.17±10.42^*^
Halogen lamp	27.06±4.16	247.02±38.37	122.62±21.92	100.24±16.72
Fluorescent lamp	27.30±6.03	255.01±50.77	120.01±12.76	106.19±11.76^*^
LED lamp	28.20±2.30	266.73±45.07	139.55±15.40^*^	101.06±14.12

Note:

# *P*<0.05 vs control group;

* *P*<0.05 vs depressive model group

## Discussion

The pathogenesis of depression is complicated, and numerous hypotheses have been drawn in the academe. The assumed monoamine neuro-transmitter is accepted as the key to study depression onset and treatment (21). The depressive symptoms is caused by the reduced of NE in the central nervous system (22). The lack of 5-HT in the central nervous system results in depression (23–24). The BDNF concentration in the serum exhibits negative correlation with Hamilton’s Depression Scale (25).

Besides, the serum BDNF levels of patients with depression was lower than those of the control group. After treatment, the serum BDNF level increased (26–27). In our experiment, the obtained results were similar to the above findings. In the experiment, the MT, 5-HT, NA, and BDNF concentrations of depressed rats were significantly lower than those of control rats, accompanied by behavioral characteristics, such as prolonged dead time, reduced sucrose preference, and slow growth of body weight and feeling of despair.

Sunlight can improve depressive symptoms. A large number of clinical depression treatments by phototherapy have been proven effective. The morbidity of depression in high-latitude areas exceeds that in the low-latitude areas (28). Residents who migrate from low-latitude areas to high-latitude area are predicted to develop depression due to the lack of sunshine (29); this finding supports the abovementioned results. Therefore, the exploration of an artificial light spectrum that can prevent depression is particularly important. After supplementary lighting by the four artificial light spectra, the depressive behaviors of depressed rats showed certain improvement. However, the intervention effect of lighting was not homogeneous, and the effect of microwave sulfur lamp was the most significant. During the behavior observation in three scores of the open-field experiment, sucrose preference, and dead-time of forced swimming, comparison with rats in the depressive model group showed significant difference. Meanwhile, no significant differences was observed in comparison with control group rats. The values of MT (*P*=0.023), 5-HT (*P*=0.018), NA (*P*=0.039), and BDNF (*P*=0.038) concentrations in rats in the microwave sulfur lamp group closely approached those of rats in the control group. Compared with the depressive model group, four indicators showed significant differences. Thus, the rats exhibited improved curiosity, regained pleasant sensation, and showed improved feeling of despair in the microwave sulfur lamp group after lighting. Moreover, the rats in the group recovered from depression.

**Fig. 3:**
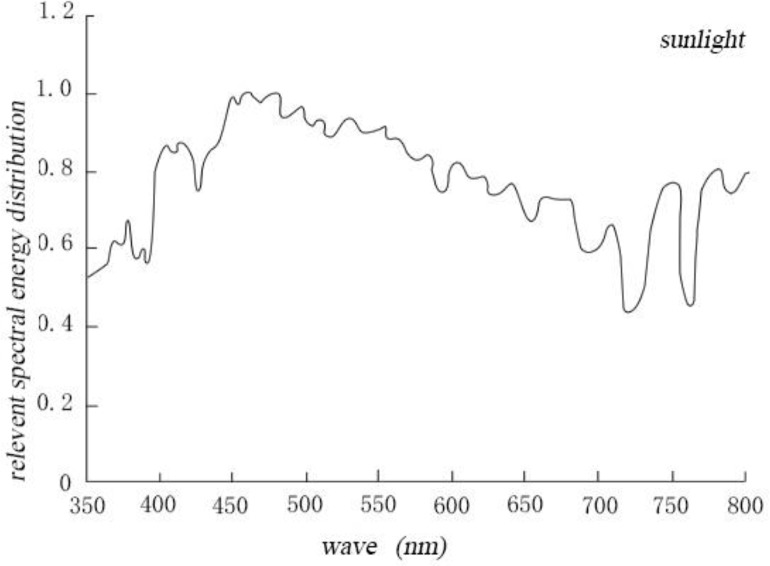
Relevant spectral energy distribution of sunlight

**Fig. 4:**
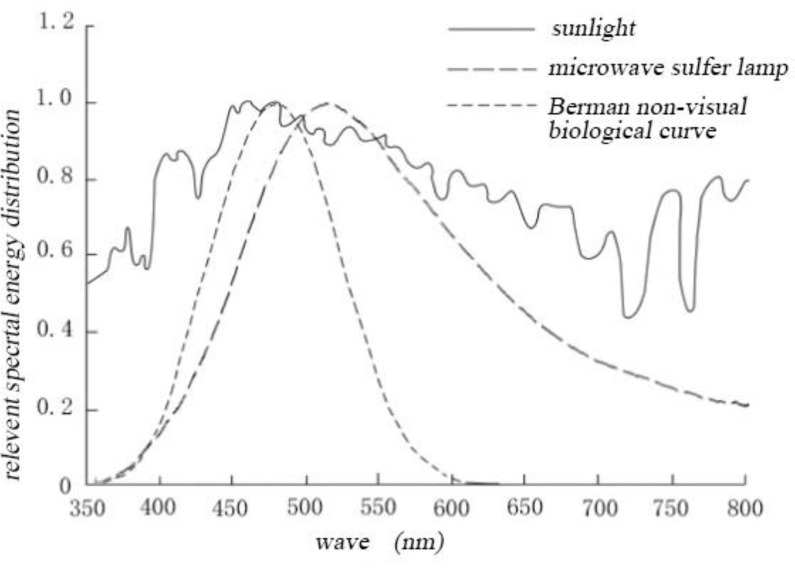
Comparison of the spectra of sulfur lamp, solar spectrum, and nonvisual biological curve

Comparing the distributions of spectral energies of four artificial light sources and sunlight, we found that within the scope of visible light, the spectra of sunlight, sulfur lamp, and halogen lamp belong to the continuous spectra. On the other hand, the fluorescent and LED lamp spectra belong to the discontinuous spectra. Compared with that of the solar spectrum (continuous spectrum), the spectral energy of the halogen lamp is concentrated in the yellow–red region, including large amount of infrared light and heat radiation. Meanwhile, the spectral energy distribution of the microwave sulfur lamp is closer to the spectral energy distribution of sunlight. By comparing the human eye nonvisual biological effect curve (30), the spectral energy distribution of the microwave sulfur lamp is basically consistent with the curve (Fig. 3 and 4)

In this study, the core symptoms of depressed human were simulated through the CUMS depressed model of rats. We provided preliminary discussions on the effects of different artificial light spectra on the behavior and hormone levels of depressed rats. However, rat retinas are not completely identical in physiological structure to human eyes. A future experimental study of humans with depression is still necessary to further verify the effects of phototherapy with different artificial light spectra on a depressed and potentially depressed population.

## Conclusion

By applying supplementary light to depressed rats by using four indoor artificial light spectra, the depressive behaviors of rats can be alleviated to a certain degree, with the effect of microwave sulfur lamp being the most significant. In our opinion, the pleasant sensation of depressed rats improved, and the depressive behaviors were corrected after the phototherapy of microwave sulfur lamp. The spectral energy distribution of microwave sulfur lamp simulated the function of sunlight, and its spectral energy distribution was consistent with the curve of human eye nonvisual biological effects. These results suggested that the simulation of sunlight by the microwave sulfur lamp is in line with the visual physiological features of human eye, thereby satisfying the sunlight requirements of patients with depression.

## Ethical considerations

Ethical issues (Including plagiarism, informed consent, misconduct, data fabrication and/or falsification, double publication and/or submission, redundancy, etc.) have been completely observed by the authors.

## References

[1] Kennedy SH (2006). A review of antidepressant treatments today. Eur Neuropsychopharm, 16:S619–S23.

[2] Holsboer F (2000). The corticosteroid receptor hypothesis of depression. Neuropsychopharmacology, 23(5):477–501. 11027914 10.1016/S0893-133X(00)00159-7

[3] Patten SB Williams JVA Lavorato DH (2017). Seasonal variation in major depressive episode prevalence in canada. Epidemiol Psychiatr Sci, 26(2):169–76. 26751782 10.1017/S2045796015001183 PMC6998685

[4] Kurlansik SL Ibay AD (2012). Seasonal affective disorder. Am Fam Physician, 86(11):1037–41. 23198671

[5] Magnusson A Boivin D (2003). Seasonal affective disorder: An overview. Chronobiol Int, 20(2):189–207. 12723880 10.1081/cbi-120019310

[6] Guzel Ozdemir P Boysan M Smolensky MH (2015). Comparison of venlafaxine alone versus venlafaxine plus bright light therapy combination for severe major depressive disorder. J Clin Psychiatry, 76(5):e645–54. 26035199 10.4088/JCP.14m09376

[7] Sahlem GL Kalivas B Fox JB (2014). Adjunctive triple chronotherapy (combined total sleep deprivation, sleep phase advance, and bright light therapy) rapidly improves mood and suicidality in suicidal depressed inpatients: An open label pilot study. J Psychiatr Res, 59:101–07. 25231629 10.1016/j.jpsychires.2014.08.015 PMC4252537

[8] Figueiro MG Plitnick BA Lok A (2014). Tailored lighting intervention improves measures of sleep, depression, and agitation in persons with alzheimer’s disease and related dementia living in long-term care facilities. Clin Interv Aging, 9:1527–37. 25246779 10.2147/CIA.S68557 PMC4168854

[9] de Bodinat C Guardiola-Lemaitre B Mocaer E (2010). Agomelatine, the first melatonergic antidepressant: Discovery, characterization and development. Nat Rev Drug Discov, 9(8):628–42. 20577266 10.1038/nrd3140

[10] Alan JG MP John CM (2010). Practice guideline for the treatment of patients with major depressive disorder, 3rd ed American Psychiatric Association, Washington DC.

[11] Lewy AJ Wehr TA Goodwin FK (1980). Light suppresses melatonin secretion in humans. Science, 210(4475):1267–69. 7434030 10.1126/science.7434030

[12] Lewy AJ Kern HA Rosenthal NE (1982). Bright artificial-light treatment of a manic-depressive patient with a seasonal mood cycle. Am J Psychiatry, 139(11):1496–98. 7137404 10.1176/ajp.139.11.1496

[13] Berson DM Dunn FA Takao M (2002). Phototransduction by retinal ganglion cells that set the circadian clock. Science, 295(5557):1070–73. 11834835 10.1126/science.1067262

[14] Rosenthal NE Sack DA Gillin JC (1984). Seasonal affective-disorder - a description of the syndrome and preliminary findings with light therapy. Arch Gen Psychiatry, 41(1):72–80. 6581756 10.1001/archpsyc.1984.01790120076010

[15] Schwartz PJ Brown C Wehr TA (1996). Winter seasonal affective disorder: A follow-up study of the first 59 patients of the national institute of mental health seasonal studies program. Am J Psychiatry, 153(8):1028–36. 8678171 10.1176/ajp.153.8.1028

[16] LeGates TA Altimus CM Wang H (2012). Aberrant light directly impairs mood and learning through melanopsin-expressing neurons. Nature, 491(7425):594–98. 23151476 10.1038/nature11673 PMC3549331

[17] Vandewalle G Schwartz S Grandjean D (2010). Spectral quality of light modulates emotional brain responses in humans. Proc Natl Acad Sci USA, 107(45):19549–54. 20974959 10.1073/pnas.1010180107 PMC2984196

[18] Warthen DM Wiltgen BJ Provencio I (2011). Light enhances learned fear. Proc Natl Acad Sci USA, 108(33):13788–93. 21808002 10.1073/pnas.1103214108 PMC3158234

[19] Kripke DF (2015). A breakthrough treatment for major depression. J Clin Psychiatry, 76(5):E660–E61. 26035202 10.4088/JCP.14com09644

[20] Kagan S Koruc Z Latifoglu G (2017). Comparison of Psychological and Physiological Changes of the Anxiety in Various Sports. Rev Cercet Interv So, 56:44–56.

[21] Castren E (2005). Opinion - is mood chemistry? Nat Rev Neurosci, 6(3):241–46. 15738959 10.1038/nrn1629

[22] Zis AP Goodwin FK (1979). Novel anti-depressants and the biogenic-amine hypothesis of depression - case for iprindole and mianserin. Arch Gen Psychiatry, 36(10):1097–107. 475543 10.1001/archpsyc.1979.01780100067006

[23] Coppen A Shaw DM Malleson A (1965). Changes in 5-hydroxytryptophan metabolism in depression. Br J Psychiatry, 111:105–7. 14261721 10.1192/bjp.111.470.105

[24] Bystritsky A Kerwin L Feusner JD (2008). A pilot controlled trial of bupropion xl versus escitalopram in generalized anxiety disorder. Psychopharmacol Bull, 41(1):46–51. 18362870

[25] Shimizu E Hashimoto K Okamura N (2003). Alterations of serum levels of brain-derived neurotrophic factor (bdnf) in depressed patients with or without antidepressants. Biol Psychiatry, 54(1):70–75. 12842310 10.1016/s0006-3223(03)00181-1

[26] Karege F Perret G Bondolfi G (2002). Decreased serum brain-derived neurotrophic factor levels in major depressed patients. Psychiatry Res, 109(2):143–48. 11927139 10.1016/s0165-1781(02)00005-7

[27] Lee HY Kim YK (2008). Plasma brain-derived neurotrophic factor as a peripheral marker for the action mechanism of antidepressants. Neuropsychobiology, 57(4):194–99. 18679038 10.1159/000149817

[28] Brown MJ Jacobs DE (2011). Residential light and risk for depression and falls: Results from the lares study of eight european cities. Public Health Rep, 126:131–40. 21563721 10.1177/00333549111260S117 PMC3072912

[29] Murase S Murase S Kitabatake M (1995). Seasonal mood variation among japanese residents of stockholm. Acta Psychiatr Scand, 92(1):51–55. 7572248 10.1111/j.1600-0447.1995.tb09542.x

[30] Berman SM Clear RD (2008). Past vision studies can support a novel human photoreceptor. Light Eng, 16(2):88–94.

